# Differences in *Albizia odoratissima* genetic diversity between Hainan Island and mainland populations in China

**DOI:** 10.3389/fpls.2024.1369409

**Published:** 2024-04-24

**Authors:** Qi An, Yuanheng Feng, Zhangqi Yang, La Hu, Dongshan Wu, Guifang Gong

**Affiliations:** ^1^ Guangxi Key Laboratory of Superior Timber Trees Resource Cultivation, Guangxi Zhuang Autonomous Region Forestry Research Institute, Nanning, Guangxi, China; ^2^ Masson Pine Engineering Technology Research Center of Guangxi, Nanning, Guangxi, China; ^3^ Key Laboratory of Central South Fast-growing Timber Cultivation of Forestry Ministry of China, Nanning, Guangxi, China

**Keywords:** *Albizia odoratissima*, EST-SSR molecular markers, genetic diversity, genetic structure, Hainan Island

## Abstract

**Background:**

This study aimed at exploring unique population genetic characteristics of *Albizia odoratissima* (Linn. f) Benth on Hainan Island to provide a scientific basis for its rational utilization and protection.

**Methods:**

It analyzed the genetic diversity and structure of 280 individuals from 10 subpopulations of *A. odoratissima* from Hainan Island and Baise City using 16 expression sequence markers - simple sequence repeat markers.

**Results:**

The genetic diversity of Hainan population (*I* = 0.7290, *He* = 0.4483) was lower than that of the Baise population (*I* = 0.8722, *He* = 0.5121). Compared with the Baise population (Nm = 2.0709, *F_ST_* = 0.1077), the Hainan Island population (Nm = 1.7519, *F_ST_* = 0.1249) exhibited lower gene flow and higher degree of genetic differentiation. Molecular variance and genetic differentiation analyses showed that the main variation originated from individuals within the subpopulation. There were significant differences in the genetic structure between Hainan and Baise populations. It grouped according to geographical distance, consistent with the Mantel test results (R^2^ = 0.77, *p* = 0.001). In conclusion, the genetic diversity of the island *A. odoratissima* population was lower than that distributed on land, the two populations exhibited obvious genetic structure differences. Both the degrees of inbreeding and genetic differentiation were higher in the island population than in the land population.

## Introduction

*Albizia odoratissima* (Linn. f) Benth is a perennial evergreen tree of *Albizia Durazz* that belongs to Leguminosae sp., Mimosaceae, commonly found in low-altitude sparse forests (Yuan et al., 2011; Ai, 2016). The plant has no spines, the twigs are initially puberulous; leaflet oblong, apex blunt; the flowers are sessile, yellowish, fragrant, capitulum arranged terminal, open panicles; pod flat, oblong, with 6-12 seeds; flowering period from April to July, fruit period from June to October. This tree species has excellent quality, rapid growth, medicinal value, is a rare fast-growing precious timber tree species (Liang, 2012; Liang, 2020; Wei et al., 2020). However, the *A. odoratissima* distribution area is shrinking due to human interference, among other reasons, and the threat of endangerment is becoming increasingly serious (Luo et al., 2020). Therefore, population genetics research is needed to effectively protect this precious species.

As a typical monsoonal evergreen broad-leaved forest species, *A. odoratissima* is mainly distributed in Guangxi, Yunnan, Guizhou, Guangdong, Sichuan, Hainan, and other provinces in China, it had also been reported in India, Vietnam, Malaysia, and other countries (Wei et al., 2020). In China, the natural population of *A. odoratissima* shows an “island-like” discontinuous distribution, spanning the South subtropical and tropical climate zones. It’s distribution area covers a variety of landforms, such as the Yunnan-Guizhou Plateau, Nanling Mountain, the Hilly area of South China, and Hainan Island Mountain. This distribution pattern is highly likely to hinder the gene exchange between *A. odoratissima* populations in different regions, resulting in rich genetic variation between them.

Hainan Island is the second-largest island in China. It was completely separated from the Asian mainland owing to the formation of the Qiongzhou Strait in the middle Pleistocene. Since then, it has faced the southernmost end of mainland China across the sea. With an area of 35400 km^2^ (Wang et al., 2022), the island has a tropical monsoon climate, rich rainfall, and sufficient heat. Known as a “species gene pool” and “natural museum,” it is also the most special distribution area of *A. odoratissima*. The Hainan population is the only *A. odoratissima* group distributed in the tropical areas of China. As a typical “continental island,” Hainan Island is an ideal place to study species genetics and evolution (Del Valle et al., 2020). For species with intermittent island and land distributions, the limited island area, long-term geographical isolation, and greatly different climatic conditions lead to obvious genetic differentiation and phenotypic differences between the populations on the island and those on the mainland during evolution. From the formation of unique or even excellent germplasm resources, research different populations of the same species distributed on both islands and the mainland can provide important clues for the intraspecific evolution that determines the early differentiation and recent spread of species (Fernández-Mazuecos and Vargas, 2011).

In general, in order to research the genetic differences between island populations and mainland populations, it is more appropriate to compare the populations in the land distribution area closest to the island. In addition, comparative studies can also be conducted using the core distribution area or origin center area populations on the mainland. The current survey results showed that there is no large-scale distribution of *A. odoratissima* populations in the Leizhou Peninsula which is closest to Hainan Islandand and the coastal areas of southern China. The Baise population, located in the northwest of Guangxi, China, is adjacent to the two distribution areas of eastern Yunnan and southwest Guizhou, is the closest to Hainan Island among the large-scale distribution areas of *A. odoratissima* found so far. The geographical distribution area of Baise is 36,000 km^2^ (Liu et al., 2017), which is similar to Hainan Island. From the perspective of biogeography, Hainan Island was once connected to Guangxi, China (Zhu, 2016). Therefore, selecting the population of Baise area as the control experimental material has good representativeness.

The research of genetic diversity is of great significance for revealing the evolutionary history of species, evaluating their survival status, and predicting their future development trends. At present, research on population genetics of species mostly relies on experimental materials from island or terrestrial populations, while there are relatively few populations from both islands and continents. As a species of important research value distributed on both islands and land, the study of population genetics of *A. odoratissima* was still limited. Simple sequence repeat (SSR) molecular marker has the advantages of abundant quantity, codominant inheritance, high polymorphism, high specificity, high universality and good repeatability (Garrido-Cardenas et al., 2018). According to their origin, they can be divided into genomic SSR (G-SSR) and expression sequence label SSR (EST-SSR). Compared with G-SSR, EST-SSR has the advantages of simple development process, low cost, high sequence conservation, and inter-species transferability (Manoj et al., 2021). The EST-SSR originates from transcription regions, which is often linked to functional genes, it has obvious advantages in population genetics research and marker-assisted breeding. In view of this, it used EST-SSR molecular markers as a tool to comprehensively evaluate the genetic diversity and structure of *A. odoratissima* populations in four regions of Hainan Island. Baise population as a representative of terrestrial population. To provide reference for the genetic evaluation of this germplasm resource and the development of high-quality genetic resources.

## Materials and methods

### Experimental materials of *A. odoratissima* population

**Figure 1:**
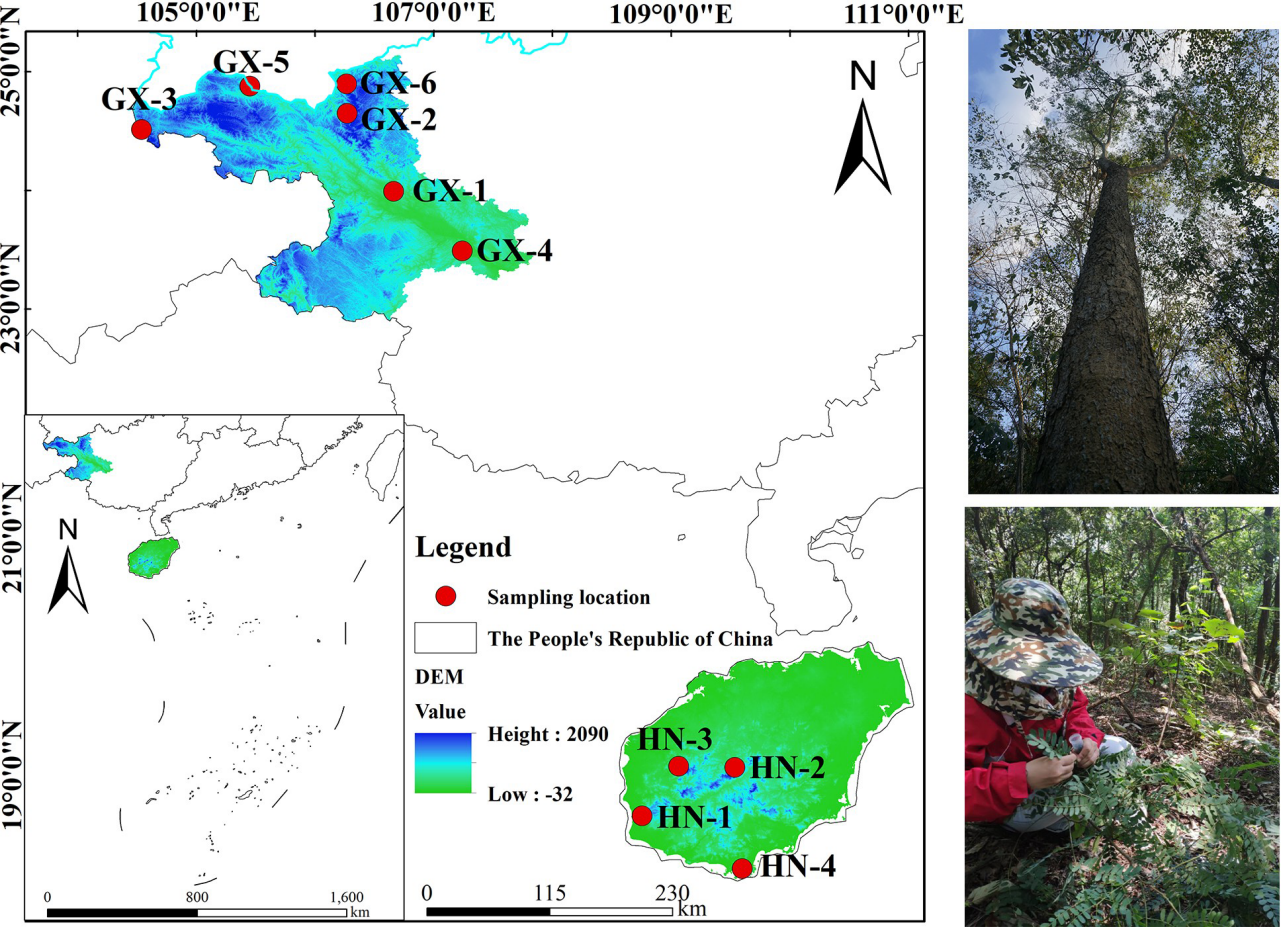
Geographical distribution of the plant materials.

Based on relevant literature, the natural population of *A. odoratissima* is mainly distributed in the west and southwest Hainan Province (**Figure 1**). Yingge Ling, Sanya City, Jianfengling, and Bawangling of Hainan Island were selected as the locations for test materials collection, representing the east, south, west, and north of the main distribution area of *A. odoratissima*. The population of Baise Prefecture in Guangxi were collected from six regions, including Youjiang District, Xilin County, Tianlin County, Tiandong County, Longlin County, and Leye County. At each collection site, according to the standard distance between plants of no less than 30 m, individual plants with normal growth, no obvious defects, diseases, and pests were selected. The top tender tree leaves from the current year were collected, silica gel was used to dry and preserve them. The collection sites for the experimental materials were evenly distributed, showing good representation. A total of 280 samples were collected from each of the ten sites (**Table 1**).

**Table 1:** Basic information of natural *A. odoratissima* populations.

Sample collection site	Subpopulation names	Subpopulation number	Longitude and latitude	Altitude(m)	Sample size	Climate
Hainan population	Jianfengling	HN-1	108°50’E	96~534	17	Tropical monsoon climate, dry rainy season obvious
			18°42’N			
	Yinggeling	HN-2	109°36’E	271~536	16	Tropical maritime monsoon climate, long summer without winter
			18°59’N			
	Bawangling	HN-3	109°08’E	126~600	25	Tropical rainforest climate, mild cli-mate, abundant rainfall
			19°15’N			
	Sanya	HN-4	109°40’E	45~260	20	Tropical maritime monsoon climate
			18°16’N			
Baise population	Chengbi Lake	GX-1	106°39’E	170~330	31	Subtropical monsoon climate
			24°00’N			
	Tianlin county	GX-2	106°10’E	350~450	30	Subtropical monsoon climate, long summer, and short winter
			24°21’N			
	Xilin county	GX-3	104°34’E	750~1100	30	Subtropical continental monsoon climate, no cold winter, and summer heat
			24°28’N			
	Tiandong county	GX-4	107°11’E	330~520	31	South Tropical monsoon climate, hot summer, mild winter, wet summer, and dry winter
			23°43’N			
	Longlin county	GX-5	105°28’E	520~580	30	Subtropical alpine climate, more obvious four seasons, no hot summer, and no cold winter
			24°50’N			
	Leye county	GX-6	106°17’E	670~710	50	Subtropical monsoon climate, summer without heat, and winter without cold
			24°51’N			

### Extraction of genomic DNA from *A. odoratissima*

Fresh plant tissues (50 – 100 mg) were ground into a powder in liquid nitrogen, and genomic DNA was extracted using an EZ – 10 Spin Column Plant Genomic DNA Purification Kit, which Providing by Sangon Biotech (Shanghai) Co., Ltd.

### SSR (simple sequence repeat)-PCR (polymerase chain reaction) amplification and detection of amplification products

Transcriptome sequencing was performed on *A. odoratissima* leaves. Considering the RNA-seq results, Novofinder software was used to search for SSR loci in the transcriptome Universal Gene of *A. odoratissima*. The SSR loci were screened following these criteria: sequence length: 18-26 bp and number of nucleotide repeats ≥ 5. 16 pairs of EST-SSR primers were selected for this study (**Table 2**). All primers were synthesized by Guangzhou Ige Biotechnology LTD, Guangzhou, China.

**Table 2:** EST-SSR primer information of *A. odoratissima*.

Primer	Repeat the primitive	Primer (5’ ~ 3’)	Tm/°C	*PIC*
AO-37	(GAA)8	F: ACGATGGAACAGTAACCGGA	59.93	0.48
		R: GTGCTGTTTGGATCCTCCAT		
AO-53	(TAT)8	F: AGGAGGAGGAGGCGTTGTAT	59.95	0.70
		R: TTCAGCTCAGCCCTGATTTT		
AO-62	(TTCG)6	F: TGCCTCACACTACACGCTTC	60.02	0.50
		R: GCGTTGCTTGAGGACTAAGG		
AO-75	(GAG)8	F: ATGCATGAGGAATGGAGGAG	60.03	0.53
		R: CCTCTCCTTATGCCTTTCCC		
AO-130	(ATT)7	F: AGCTCTAAAAGCAGGTGGCA	59.83	0.40
		R: GCCTGTGTCATCATCGCTTA		
AO-133	(GCC)7	F: AGGATTAAGCAAAGCGCTGA	59.96	0.43
		R: CGGAGTTGGCAGTGGATATT		
AO-141	(AGA)7	F: AGGAAGTGTCCAACTGGGTG	59.98	0.46
		R: GGCGTCTTCGCTATTCAAAG		
AO-146	(CAG)7	F: ATCTGAGATGGCTTGTTGGG	59.98	0.54
		R: TTTGCTGCATATCTCGTTGC		
AO-166	(ATG)7	F:TTCGTGGAATCGATCAATCA	59.93	0.47
		R: TGGCTCCAACATCCCTTAAC		
AO-184	(TCTT)5	F: TGGGGGAACAGTGGTTATGT	59.98	0.50
		R: TCTCTGTTCGTCATTCGTCG		
AO-188	(TTAA)5	F: GCTCCCAATATCCATGTGCT	59.92	0.61
		R: TGAAGGATATCACCGCATCA		
AO-189	(TTAA)5	F: ATGCAGGTTGCAATCAATCAA	59.98	0.60
		R: TTTGGGAATTGGGGATTACCA		
AO-194	(AAGA)5	F: CTTCACCGGATCTAGGACCA	59.97	0.08
		R: ATTCGGAAACGAACCAGTTG		
AO-199	(TACA)5	F: TCATCAATGTGCTTCCCAAA	59.89	0.57
		R: AGCTCAAGCAGCTCAGGAAC		
AO-210	(GAAA)5	F: GTTTCCATGGTGATATGGGC	60.07	0.28
		R: ATGTCCCAGAGAATGCCAAG		
AO-217	(GCAG)5	F: TCTCCCATCAAAATCCAAGC	60.00	0.58
		R: CTTGGAGAATCCCATCGAAA		

The PCR system was 10.00 μL, including template DNA (1.00 μL; 50 ng/stock), 10 × buffer (1.00 μL; 1 × Buffer), dNTPs (including Mg^2+^) (0.20 μL; 0.2 mmol/L), positive and negative primers (0.25 μL; 0.25 μmol/L), Taq DNA enzyme (0.10 μL 0.25 U/stock), and ddH_2_O (7.20 μL). The PCR procedure was as follows: 94°C pre-denaturation for 4 min; 25 cycles of 94°C denaturation for 15 s, 58°C annealing for 15 s, 72°C extension for 30 s; extension at 72°C for 20 min; and storage at 12°C. The amplified products were separated using 8% polyacrylamide gel electrophoresis.

### Data analysis

POPGENE 32 (Yeh et al., 1999) software was used to calculate the following genetic parameters: average number of alleles (*A*), observed number of alleles (*Na*) (Fang et al., 2018), effective number of alleles (*Ne*) (Hartl and Clark, 1989), Shannon’s Information Index (*I*), and Nei’s gene diversity index (*H*) (Shannon and Weaver, 1949; Shannon et al., 1950), observed heterozygosity (*Ho*), expected heterozygosity (*He*) (Nei, 1973), and Wright’s fixation index (*F*) could be obtained from the formula 1-*F_IT_* = (1-*F_IS_*) (1-*F_ST_*), used to determine *F_IS_*, *F_IT_*, and genetic differentiation index (*F_ST_*) (Wright, 1978). Nei’s standard genetic distance (*GD*) (Nei, 1972), genetic identity, gene flow (Nm) (Slatkin and Barton, 1989), and genetic differentiation coefficient (*G_ST_*).

STRUCTURE 2.3.4 (Pritchard et al., 2000) software was used to perform Bayesian clustering analysis on individuals from different populations. Subpopulation grouping was determined based on the optimal K value obtained via STRUCTURE Harvester (https://taylor0.biology.ucla.edu/structureHarvester) analysis.

The polymorphism information content (*PIC*) for each locus was calculated using the online program *PIC* calc (Botstein et al., 1980).

Arlequin 3.5 (Excoffier and Lischer, 2010) software was used to analyze the molecular variation at different levels within and between populations.

Mantel test was performed using GenAlEx 6.5 (Peakall and Smouse, 2012). The Mantel test was used to assess the correlation between geographic distance and genetic distance.

The UPGMA (unweighted pair-group method with arithmetic means) clustering tree was constructed using NTSYS PC (Rohlf, 2000) software based on Nei’s standard genetic distance.

The ape (Paradis and Schliep, 2019) and ggplot 2 (Wickham, 2016) packages of R 2.3.4 were analyzed for principal coordinates analysis (PCoA).

## Results

### Screening of EST-SSR primers for *A. odoratissima*

The results of the previous study showed that among the 243 pairs of developed primers of *A. odoratissima*, the effective amplification rate in *Albizia odoratissima Albizia procera, Albizzia falcataria, Acacia melanoxylon* and *Erythrophloeum fordii* was 63.79%, 33.75%, 45.68%, 41.56% and 14.81% respectively, the polymorphism ratio in them was 23.87%, 12.20%, 9.01%, 3.96% and 2.78% respectively (An et al., 2022). A total of 16 materials were randomly selected from various subpopulations of *A. odoratissima* in Guangxi and Hainan, the polymorphisms of 155 pairs of effective primers were re-detected. Finally, 16 pairs of primers with good generality, high polymorphism and clear bands were selected for the genetic diversity study of the germplasm resources of *A. odoratissima.* The polymorphism information content was analyzed, as shown in **Table 2**. The effective amplification rate of 16 selected primers in *Albizia procera*, *Albizia falcataria* and *Acacia melanoxylon* was 50%, and the proportion of polymorphic primers in effective primers was 50%, 12.5% and 12.50%, respectively. Only one pair of primers can have effective amplification rate and no polymorphism in *Erythrophleum fordii*. It can be seen that the polymorphic information content of the 16 SSR primers used in this experiment ranged from 0.08 to 0.70, with an average value of 0.48. The 16 pairs of primers selected were all medium-high polymorphic primers, except for AO-194, which was a low polymorphic primer. Indicating that the screened primers could be used for the genetic diversity study of the germplasm resources of *A. odoratissima*. It had certain universality in *Albizia procera*, *Albizia falcataria*, *Acacia melanoxylon* and *Erythrophleum fordii*.

### Genetic diversity of *A. odoratissima* population

A total of 52 alleles were amplified by 16 SSR loci in 280 individuals from 10 A*. odoratissima* subpopulations (**Table 3**), the number of alleles detected at each site ranged from 2.00 to 5.00.

**Table 3:** Genetic diversity in 10 populations of *A. odoratissima*.

population	*N*	*Na*	*Ne*	*Ho*	*He*	*I*
HN-1	41	2.56	1.82	0.2246	0.4150	0.6537
HN-2	38	2.38	1.84	0.2126	0.4132	0.6434
HN-3	38	2.38	1.85	0.2095	0.4092	0.6452
HN-4	39	2.44	1.7	0.1869	0.3820	0.6088
Population level	43	2.69	1.96	0.2075	0.4483	0.7290
GX-1	46	2.88	1.96	0.2542	0.4473	0.7474
GX-2	47	2.94	2.01	0.2786	0.4476	0.7378
GX-3	47	2.94	2.04	0.3737	0.4855	0.7927
GX-4	48	3.00	2.06	0.2555	0.4600	0.7830
GX-5	44	2.75	1.98	0.2744	0.4436	0.7261
GX-6	46	2.88	2.12	0.2591	0.4715	0.7872
Population level	52	3.25	2.24	0.2800	0.5121	0.8722
Species level	52	3.25	2.36	0.2615	0.5366	0.9079

N, Total number of alleles; Na, Observed number of alleles; Ne, Effective number of alleles; Ho, Observed Heterozygosity; He, Expected Heterozygosity; I, Shannon’s Information Index; H, Nei’s gene diversity index.

In terms of gene abundance (**Table 3**), 43 and 52 alleles were detected at 16 SSR loci in Hainan and Baise populations, respectively. Compared with the Baise population, the Hainan population had allele deletions at 50.00% of loci and no specific alleles (**Table 4**). The average number of observed alleles (*Na*) in Hainan population was 2.69, which was 17.23% lower than that in Baise population. The average number of effective alleles (*Ne*) in Hainan population was 1.96, which was 12.50% lower than that in the Baise population. To some extent, it showed that after geographical isolation from the mainland, Hainan population were greatly limited in introducing new alleles through gene communication.

**Table 4:** Allele frequencies of *A. odoratissima* populations from Hainan and Baise.

Allele	Hainan population					Baise population				
	A	B	C	D	E	A	B	C	D	E
AO-39	34.87%	65.13%				29.95%	53.81%	16.24%		
AO-53	0.67%	2.00%	74.00%	23.33%		52.01%	14.07%	6.28%	16.08%	11.56%
AO-62	28.57%	51.95%	19.48%			30.25%	61.50%	8.25%		
AO-75	22.08%	49.35%	28.57%			10.05%	68.81%	21.13%		
AO-130	22.67%	63.33%	14.00%			11.36%	81.31%	7.32%		
AO-133		24.67%	75.33%			2.99%	78.61%	18.41%		
AO-141		98.08%	1.92%			26.41%	51.54%	22.05%		
AO-146	38.67%	26.67%	34.67%			19.49%	27.95%	52.56%		
AO-166	4.86%	36.11%	59.03%			1.02%	35.03%	63.96%		
AO-184	14.67%	85.33%				45.75%	41.75%	11.75%	0.75%	
AO-188	11.46%		10.42%	78.13%		42.94%	6.18%	40.59%	10.29%	
AO-189	7.29%		35.42%	57.29%		45.86%	2.37%	30.77%	21.01%	
AO-194	94.23%	5.77%				99.75%	0.25%			
AO-199	30.92%	15.13%	53.95%			40.86%	20.05%	38.83%	0.25%	
AO-210	49.36%	50.64%				25.00%	75.00%			
AO-217	59.46%	17.57%	22.97%			33.60%	43.55%	22.85%		

The average *He*, *Ho*, *I*, and *H* values of the Hainan *A. odoratissima* population were 0.4483, 0.2075, 0.7290, and 0.4452, respectively (**Table 3**). which were 87.54%, 74.10%, 83.58%, and 87.16% lower than those of Baise population, respectively. Thus, genetic complexity was lower in the Hainan population than in the Baise population. In the four subpopulations of Hainan, *Ho* ranged from 0.1869 to 0.2246, *He* ranged from 0.3820 to 0.4150, *I* ranged from 0.6088 to 0.6537, and *H* ranged from 0.3706 to 0.4021. In the six subpopulations of Baise, *Ho* ranged from 0.2542 to 0.3737, *He* ranged from 0.4436 to 0.4855, *I* ranged from 0.7261 to 0.7927, and *H* ranged from 0.4357 to 0.4765. The *He*, *Ho*, *I* and *H* values of the four Hainan subgroups were all lower than those of the six subgroups of Baise.

There were certain differences in allele frequencies between the two populations at different loci (**Table 4**), with a maximum of 67.84%, indicating that the two populations have different evolutionary directions due to differences in natural environment.

### Population genetic differentiation and genetic variation

The Nm (0.9976) between the two populations of *A. odoratissima* was lower than that of Hainan (Nm=1.7559) and Baise (Nm=2.0709), respectively. This indicates that gene exchange between the two populations was low, but the degree of gene exchange within the population was relatively high.

The *Ho* values of both populations were lower than the *He* value, the inbreeding coefficients of each subgroup were greater than zero, indicating that both populations had a certain degree of homozygosity and inbreeding. Among them, the inbreeding coefficient of the GX-3 subgroup was close to zero, showing that it was closest to the Hardy Weinberg equilibrium.

From the *F* statistical data (**Table 5**), it canould be seen that the *F_ST_* and *G_ST_* values of the *A. odoratissima* population in Hainan Province were 0.1249 and 0.1000, respectively, while the population in Baise City were 0.1077 and 0.0980, respectively. Therefore, the genetic differentiation of the Hainan population was higher than that of the Baise population. The genetic variation of the two populations mainly comes from subpopulations.

**Table 5:** Analysis of molecular variance (AMOVA) for population of *A. odoratissima*.

Population	Source of variation	d.f.	Sum of squares	Variance components	Percentage of variation	Fixation Indices
Hainan population	Among populations	3	33.255	0.20478 Va	8.14	*F_IS_* =0.4671
	Among individuals Within populations	74	235.649	0.87427 Vb	34.76	*F_ST_* =0.1249
	Within individuals	78	112	1.43590 Vc	57.09	*F_IT_* =0.5336
	Total	155	380.904	2.51495	100	*G_ST_* =0.1000
Baise population	Among populations	5	134.244	0.35007 Va	11.47	*F_IS_* =0.3768
	Among individuals Within populations	196	686.053	0.79840 Vb	26.16	*F_ST_* =0.1077
	Within individuals	202	384.5	1.90347 Vc	62.37	*F_IT_* =0.4439
	Total	403	1204.797	3.05194	100	*G_ST_* =0.0980
Total population	Among populations	9	305.738	0.55071 Va	17.26	*F_IS_* =0.4130
	Among individuals Within populations	270	946.005	0.86347 Vb	27.06	*F_ST_* =0.2004
	Within individuals	280	497.500	1.77679 Vc	55.68	*F_IT_* =0.5306
	Total	559	1749.243	3.19096	100	*G_ST_* =0.1838

Df, Degree of freedom.

Further analysis of variance was performed at the population, subpopulation and individual levels (**Table 5**). Both populations exhibit within individuals > among individuals Within populations> among populations. It indicated that the genetic variation of *A. odoratissima* mainly came from the variation between individuals.

### Genetic structure of *A. odoratissima* population

**Figure 2:**
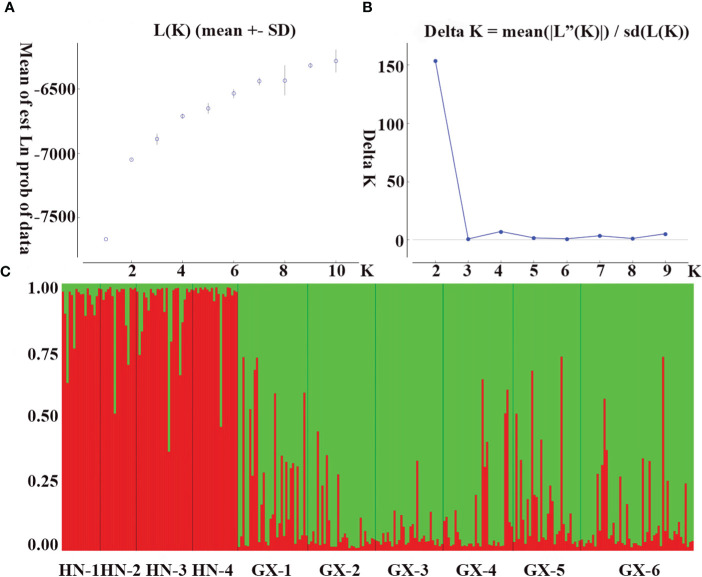
Population genetic structure. **(A)** Mean log-likelihood [Ln(K)±SD] against the number of K; **(B)** Relations between the rational groups number K and Estimated value ΔK. **(C)** Genetic structural plot of 10 *A. odoratissima* subpopulations based on structure analysis (Each individual is represented by a single vertical bar, which is partitioned into two different colors).

**Figure 3:**
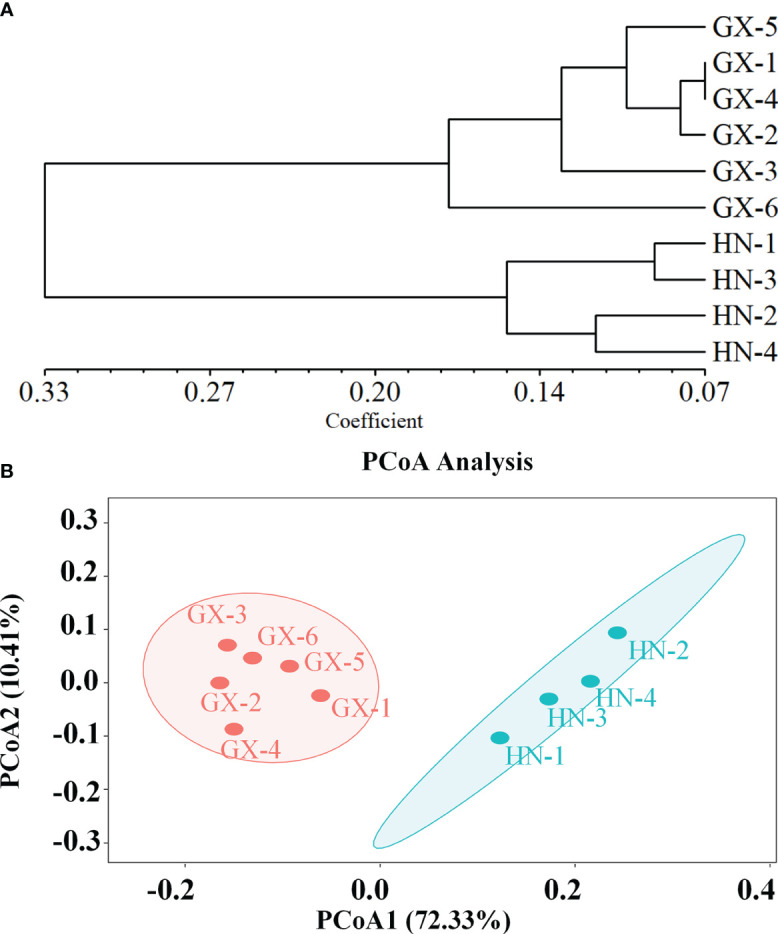
**(A)** Genetic divergence among 10 subpopulations of *A. odoratissima* based on UPGMA clustering analysis. **(B)** Principal coordinate analysis (PCoA) of 10 *A. odoratissima* subpopulations.

**Figure 4:**
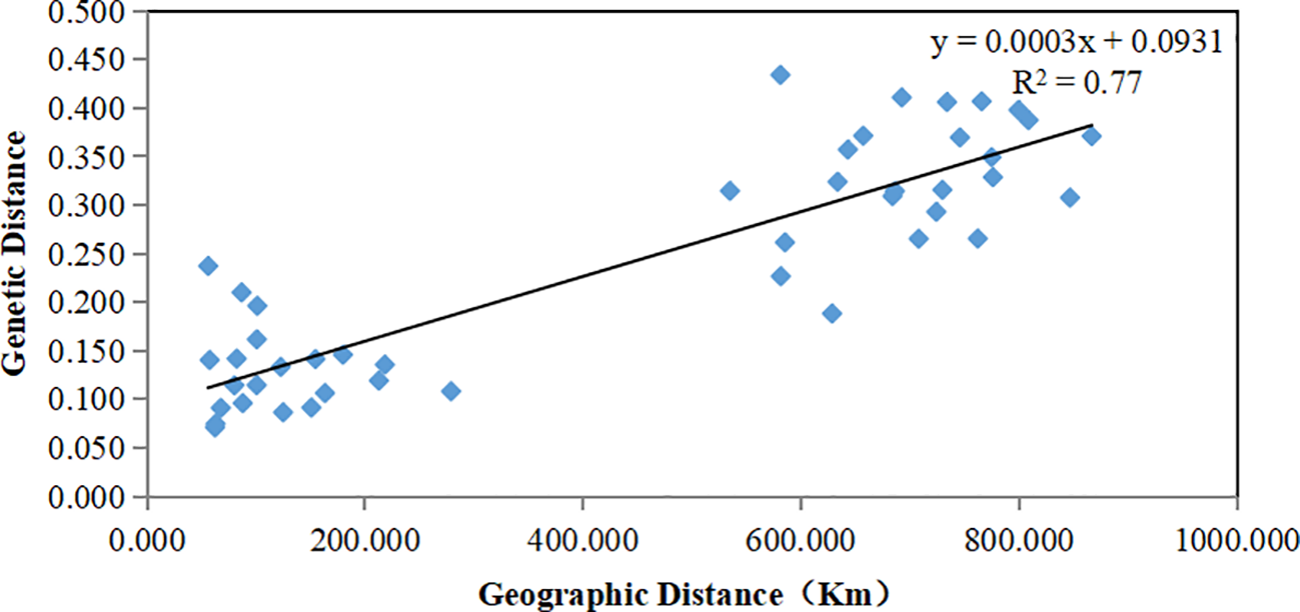
Mantel between genetic distance and geographical distance of 10 *A. odoratissima* subpopulations.

The genetic structure of *A. odoratissima* was predicted using STRUCTURE software. When K = 2, the ΔK value was the highest (**Figures 2A, B**), indicating that it was most reasonable to divide 280 samples into two groups of independent evolutionary units (**Figure 2C**). Based on the Q value, it also plotted the cluster member proportion of each subpopulation when K = 2. The four Hainan subpopulations were composed of individuals with red clusters, and the six Baise subpopulations were composed of individuals with green clusters. The results were consistent with those of UPGMA cluster analysis (**Figure 3A**) and PCoA (**Figure 3B**) based on *GD*. This shows obvious differences in genetic structure between the Hainan and Baise populations of *A. odoratissima*. It further analyzed the correlation between *GD* and geographic distance using Mantel test (**Figure 4**). *GD* was significantly correlated with geographical distance (R^2^ = 0.77, *p* = 0.001), indicating an obvious geographical origin structure or main distance isolation among the investigated populations.

## Discussion

### Genetic diversity of the *A. odoratissima* population

The genetic diversity of trees directly affects their evolutionary potential and adaptability to environmental changes. Higher genetic diversity indicates stronger evolutionary potential and ability to match the environment (Xia, 1999; Booy et al., 2000). The genetic diversity of a species is closely related to its geographical distribution, mating system, gene flow, genetic drift, and human interference (Jaenike, 1973; Fan et al., 2019; Jiang et al., 2019).

This study revealed that the genetic abundance and complexity of the *A. odoratissima* population in Hainan were lower than those of the Baise population. This phenomenon that the genetic diversity of island population was lower than that of land population also exists in some species such as *Mussaenda kwangtungensis* (Guangdong) (Hufford et al., 2014; Shi et al., 2020; Hunt et al., 2022). Long-term geographic isolation is probably the main reason explaining the lower genetic diversity of the Hainan population. This phenomenon is related to factors, such as Nm blocking, genetic drift, and small population bottleneck effect allele loss (Avise, 1996; Rieseberg and Swensen, 1996; Balmford et al., 1997).

Compared to the Baise population, allele deletion occurred in more than half of the loci in the Hainan population. It found that the two groups had lower levels of genetic communication. Indicating that the existence of geographical isolation makes it difficult for Hainan population to acquire new genes through gene exchange from land population. In additionthe, population size on Hainan Island was small, which may complicate gene mutation fixing in this population. The geographical distribution area of Baise population is close to Hainan, located at the junction of Guangxi, Guizhou, and Yunnan provinces in China, adjacent to Vietnam to the south. The population size of Baise was large, there were fewer gene exchange barriers between nearby populations, which helps to accept new genes through gene exchange and fix mutated genes. It may also be the main factor why a unique haplotype was not found in the Hainan population. It is also the reason that genetic diversity of Hainan population is lower than that of Baise population.

The genetic diversity of species dominated by outbreeding is generally higher than that of species dominated by self - breeding, the inter - population variation of the former accounts for more than 50% of the total variation (Gao and Li, 2008; Lu et al., 2021). The genetic diversity of the total population (*Ho* = 0.2615, *He* = 0.5366) was higher than that of wild soybean (*Ho* = 0.0310, *He* = 0.4260) (He et al., 2012), as well as the average of the major self - crossing species (*Ho* = 0.1200, *He* = 0.3030) (Hamrick and Godt, 1996). In addition, 18.38% of the genetic variation existed among the subpopulations, it can be reasonably inferred that the breeding system was dominated by out - crossing. The blossoms of *A. odoratissima* are light yellow, fragrant, terminal, and dispersed panicles, conforming to the characteristics of insect pollination. Affected by the flight distance of insects, the ability of the wild population to spread pollen among subpopulations was limited by distance, terrain, and other factors (Kwon and Morden, 2002).The Hainan population is more about gene exchange among subpopulations on the island, the richness of genetic diversity is lower than that of the Baise population located at the border of the three provinces and in the core distribution area.

### Genetic differentiation and genetic structure of the *A. odoratissima* population

The genetic structure of a population is the result of the interaction between ecological and genetic processes. Related research is of great significance for understanding population genetic characteristics and dynamics, and developing effective protection measures (Leberg, 1990; Cheng et al., 2020). There were significant differences in genetic structure between the Hainan and Baise populations, the genetic variation mainly came from within the population. The coefficient of genetic differentiation between the two populations (*F_ST_* = 0.2004) was significantly higher than that of Hainan and Baise populations (*F_ST_* = 0.1249 and *F_ST_* = 0.1077). In the cluster analysis, six Baise subpopulations were grouped into one cluster, and four Hainan subpopulations were grouped into another cluster. Hainan had different geographical, topographic, water, thermal conditions from Baise. Under the effect of selection, the Hainan population had taken a different evolutionary direction from that of the Baise population in order to accommodative the local environment. The existence of geographical isolation resulted in genetic differentiation between Hainan and mainland populations, while the outcrossing breeding system and the characteristics of insect pollination further intensified the genetic differentiation between island and land populations. In the wild, the *A. odoratissima* are mainly propagated by seeds, when the seeds mature, the pods crack. Affected by seed weight, wind speed, and media, the seeds generally fall not far from the mother plants, can also spread to further places along rivers. Nevertheless, the long-term geographical isolation makes it difficult for the Hainan population to rely on water flow or some media to break through the geographical isolation barrier and smoothly communicate genes with the land population. The degree of pollen dispersal among long-distance subpopulations was also lower, therefore, the Hainan population is more about gene exchange among subpopulations on the island. The isolation of the sea resulted in a lower degree of gene exchange between island and land populations than within the population, making the genetic differentiation between island and land populations more pronounced (Wright, 1951; Grant, 1986; Manel, 2003; Saro et al., 2015).

In addition, the degree of inbreeding and genetic differentiation were higher in Hainan than in Baise, consistent with the conclusion of Chen et al (Chen et al., 2008). on *Ficus pumila*. The small size and scattered distribution of the natural *A. odoratissima* population may be underlying reasons for inbreeding in this population. The resources of *A. odoratissima* were abundant in Hainan, their distribution range was limited, and the population is smaller than that in Baise. There are many tall mountains on the island, creating a mountain isolation effect greater than natural transportation effect (Frankham, 1998). It prevents gene exchange among subpopulations and facilitating inbreeding between individuals. The existence of inbreeding reduces population heterozygosity and further increases genetic differentiation of the populations.

### Conservation and utilization of *A. odoratissima* genetic resources

Some alleles were found to exist in only a few subpopulations, there was abundant genetic variation among individuals within the populations. However, due to human interference, the number of its natural population is decreasing dramatically. In view of this, it can further expand the select range of superior trees, increase their number, and establish seed orchards with a wider genetic basis than the original. Establishing seed orchards with a wider genetic basis can better preserve *A. odoratissima* resources. Seeds with better genetic quality can also be produced to establish high -quality *A. odoratissima* plantations to meet production needs and increase the breeding value of high-quality genetic resources. In addition, the study found that different subpopulations had different levels of genetic diversity, there were rare alleles with allele frequencies less than 0.01 in the *A. odoratissima* population, indicating that the rare alleles carrying certain genetic variants in the population were likely to exist in only one or a few individuals, these genetic variants were facing loss. Therefore, it is necessary to select some subpopulations with high genetic diversity and establish nature reserves to protect them in situ.

## Conclusions

This article used EST-SSR molecular markers as a research tool to comprehensively evaluate the genetic diversity and structure of two *A. odoratissima* populations from Hainan Island and Baise, Guangxi, China. The genetic variation of the population mainly comes from individual to individual. The *A. odoratissima* population distributed in Hainan Island has lower genetic diversity than the Baise population, the degree of genetic differentiation. The Hainan Island *A. odoratissima* population had a 50% loss of alleles compared to the Baise population, and there are no specific alleles. There were significant differences in the genetic structure of the two populations, with different evolutionary directions. These findings have improved our current understanding of the genetic diversity and population genetic structure of *A. odoratissima*. It can provide a theoretical basis for the improvement of seed orchard, further development, utilization, and protection of this species.

## Data availability statement

The datasets presented in this study can be found in onlinerepositories. The names of the repository/repositories and accessionnumber(s) can be found in the article/**Supplementary Material**.

## Author contributions

QA: Conceptualization, Data curation, Formal analysis, Investigation, Validation, Writing – original draft, Writing – review & editing. YF: Conceptualization, Methodology, Validation, Writing – review & editing. ZY: Conceptualization, Data curation, Funding acquisition, Project administration, Validation, Writing – review & editing. LH: Investigation, Validation, Writing – review & editing. DW: Investigation, Validation, Writing – review & editing. GG: Formal analysis, Methodology, Writing – review & editing.

## References

[B1] Ai T. M. (2016). Annals of medicinal plants of China Vol. 5 (Beijing: Peking University Medical Press), 143.

[B2] An Q. Feng Y. H. Yang Z. Q. Hu L. (2022). EST-SSR marker development and interspecific generality of *Albizzia odoratissima* . Guihaia 42 (8), 1374–1382.

[B3] Avise J. C. (1996). Conservation genetics: case histories from nature. J. Appl. Ecol. 78.

[B4] Balmford A. Avise J. C. Hamrick J. L. (1997). Conservation genetics: case histories from nature. J. Appl. Ecol. 34 (3), 829. doi: 10.2307/2404927

[B5] Booy G. Hendriks R. J. J. Smulders M. J. M. van Groenendael J. M. Vosman B. (2000). Genetic diversity and the survival of populations. Plant Biol. 2, 379–395. doi: 10.1055/s-2000-5958

[B6] Botstein D. White R. L. Skolnick M. Davis R. W. (1980). Construction of a genetic linkage map in man using restriction fragment length polymorphisms. Am. J. Hum. Genet. 32, 314–331. 6247908 PMC1686077

[B7] Chen Y. Shi M. Ai B. Gu J. Chen X. (2008). Genetic variation in island and mainland populations of *Ficus pumila* (Moraceae) in eastern Zhejiang of China. Symbiosis 45, 37–44.

[B8] Cheng J. Kao H. Dong S. (2020). Population genetic structure and gene flow of rare and endangered *Tetraena mongolica* Maxim. revealed by reduced representation sequencing. BMC Plant Biol. 20, 391. doi: 10.1186/s12870-020-02594-y 32842966 PMC7448513

[B9] Del Valle J. C. Herman J. A. Whittall J. B. (2020). Genome skimming and microsatellite analysis reveal contrasting patterns of genetic diversity in a rare sandhill endemic (*Erysimum teretifolium*, Brassicaceae). PloS One 15, e0227523. doi: 10.1371/journal.pone.0227523 32459825 PMC7252598

[B10] Excoffier L. Lischer H. E. (2010). Arlequin suite ver 3.5: a new series of programs to perform population genetics analyses under Linux and Windows. Mol. Ecol. Resour. 10, 564–567. doi: 10.1111/j.1755-0998.2010.02847.x 21565059

[B11] Fan J. J. Zhang X. P. Liu K. Liu H. J. Zhang L. Wang X. P.. (2019). The population genetic diversity and pattern of *Pteroceltis tatarinowii*, a relic tree endemic to China, inferred from SSR markers. Nordic J. Bot. 37 (2). doi: 10.1111/njb.01922

[B12] Fang S. S. Xie X. F. Qi J. M. Zhang L. M. Xu J. T. Lin L. H.. (2018). Universality of simple sequence repeat (SSR) markers from Cotton (*Gossypium hirsutum*) to Kenaf (*Hibiscus cannabinus*). Chin. J. Trop. Crops 39, 1373–1382. doi: 10.3969/j.issn.1000-2561.2018.07.017

[B13] Fernández-Mazuecos M. Vargas P. (2011). Genetically Depauperate in the Continent but Rich in Oceanic Islands: *Cistus monspeliensis* (Cistaceae) in the Canary Islands. PloS One 6, e17172. doi: 10.1371/journal.pone.0017172 21347265 PMC3038934

[B14] Frankham R. (1998). Inbreeding and extinction: Island population. Conserv. Biol. 12, 665–675. doi: 10.1046/j.1523-1739.1998.96456.x

[B15] Gao J. Li Q. M. (2008). Genetic diversity of natural populations of *Acacia pennata* in Xishuang-banna, Yunnan. Biodiversity Sci. 16, 271–278. doi: 10.3321/j.issn:1005-0094.2008.03.009

[B16] Garrido-Cardenas J. A. Mesa-Valle C. Manzano-Agugliaro F. (2018). Trends in plant research using molecular markers. Planta 247, 543–557. doi: 10.1007/s00425-017-2829-y 29243155

[B17] Grant V. (1986). The evolutionary process: a critical study of evolutionary theory. Stud. Hist Phil Sci. 17, 65–98.

[B18] Hamrick J. L. Godt M. J. W. (1996). Effects of life history traits on genetic diversity in plant species. Philos. Trans. R. Soc Lond 351, 1291–1298. doi: 10.1098/rstb.1996.0112

[B19] Hartl D. L. Clark A. G. (1989). Principles of population genetics. 2nd ed (Sunderland, MA: Sinauer Associates).

[B20] He S. Wang Y. Volis S. Li D. Yi T. (2012). Genetic diversity and population structure: implications for conservation of wild soybean (Glycine soja Sieb. et Zucc) based on nuclear and chloroplast microsatellite variation. Int. J. Mol. Sci. 13, 12608–12628. doi: 10.3390/ijms131012608 23202917 PMC3497291

[B21] Hufford K. M. Mazer S. J. Hodges S. A. (2014). Genetic variation among mainland and island populations of a native perennial grass used in restoration. AoB Plants 6, plt055. doi: 10.1093/aobpla/plt055 24790118 PMC3966692

[B22] Hunt D. A. G. A. DiBattista J. D. Hendry A. P. (2022). Effects of insularity on genetic diversity within and among natural populations. Ecol. Evol. 12, e8887. doi: 10.1002/ece3.8887 35571757 PMC9077629

[B23] Jaenike J. R. (1973). A steady state model of genetic pomorphism on islands. Am. Nat. 107, 793–795. doi: 10.1086/282878

[B24] Jiang H. Long W. Zhang H. Mi C. Zhou T. Chen Z. (2019). Genetic diversity and genetic structure of *Decalobanthus boisianus* in Hainan Island, China. Ecol. Evol. 9, 5362–5371. doi: 10.1002/ece3.5127 31110685 PMC6509374

[B25] Kwon J. A. Morden C. W. (2002). Population genetic structure of two rare tree species (*Colubrina oppositifolia* and *Alphitonia ponderosa*, Rhamnaceae) from Hawaiian dry and mesic forests using random amplified polymorphic DNA markers. Mol. Ecol. 11, 991–1001. doi: 10.1046/j.1365-294X.2002.01497.x 12030978

[B26] Leberg P. L. (1990). Genetic considerations in the design of introduction programs transactions of the north American wildlife. Natural Resource Conf. 55, 609–619.

[B27] Liang S. H. (2020). Analysis of technical measures for building *Albizia odoratissima* forest in Yachang forest area. Agric. Technol. 40, 87–88. doi: 10.19754/j.nyyjs.20201030028

[B28] Liang S. Y. (2012). Sylva guangxigensis. Volume I (Beijing: China Forestry Publishing House), 382.

[B29] Liu X. Liu Z. Zhang Y. Jiang B. (2017). The effects of floods on the incidence of bacillary dysentery in baise (Guangxi province, China) from 2004 to 2012. Int. J. Environ. Res. Public Health 14, 179. doi: 10.3390/ijerph14020179 28208681 PMC5334733

[B30] Lu J. Zhang Y. Diao X. Yu K. Dai X. Qu P.. (2021). Evaluation of genetic diversity and population structure of *Fragaria nilgerrensis* using EST-SSR markers. Gene 796-797, 145791. doi: 10.1016/j.gene.2021.145791 34175390

[B31] Luo Q. F. Hu L. Tan J. H. Jia J. Feng Y. H. Yang Z. Q. (2020). Phenotypic diversity of seeds of *Albizia odoratissima* from Baise district. J. For. Environ. 40, 62–67. doi: 10.13324/j.cnki.jfcf.2020.01.009

[B32] Manel S. (2003). Landscape genetics: combining landscape ecology and population genetics. Trends Ecol. Evol. 18, 189–197. doi: 10.1016/S0169-5347(03)00008-9

[B33] Manoj K. G. Ravindra D. Sabarinathan S. Gayatri G. Goutam K. D. Pallabi P.. (2021). Microsatellite markers from whole genome and transcriptomic sequences. Bioinf. Rice Res., 387–412. doi: 10.1007/978-981-16-3993-7_18

[B34] Nei M. (1972). Genetic distance between populations. Am. Nat. 106, 283–292. doi: 10.1086/282771

[B35] Nei M. (1973). Analysis of gene diversity in subdivided populations. Proc. Natl. Acad. Sci. U.S.A. 70, 3321–3323. doi: 10.1073/pnas.70.12.3321 4519626 PMC427228

[B36] Paradis E. Schliep K. (2019). ape 5.0: an environment for modern phylogenetics and evolutionary analyses in R. Bioinformatics 35, 526–528. doi: 10.1093/bioinformatics/bty633 30016406

[B37] Peakall R. Smouse P. E. (2012). GenAlEx 6.5: genetic analysis in Excel. Population genetic software for teaching and research an update. Bioinformatics 28, 2537–2539. doi: 10.1093/bioinformatics/bts460 22820204 PMC3463245

[B38] Pritchard J. K. Stephens M. Donnelly P. (2000). Inference of population structure using multilocus genotype data. Genetics 155, 945–959. doi: 10.1093/genetics/155.2.945 10835412 PMC1461096

[B39] Rieseberg L. H. Swensen S. M. (1996). Conservation genetics of endangered island plants. Conservation Genetics Case Histories from Nature. doi: 10.1007/978-1-4757-2504-9

[B40] Rohlf F. J. (2000). NTSYS-pc numerical taxonomy and multivariate analysis system solutions manual. Am. Stat. 41, 330.

[B41] Saro I. González-Pérez M. A. García-Verdugo C. Sosa P. A. (2015). Patterns of genetic diversity in *Phoenix canariensis*, a widespread oceanic palm (species) endemic from the Canarian archipelago. Tree Genet. Genomes 11, 1–13. doi: 10.1007/s11295-014-0815-0

[B42] Shannon C. E. Weaver W. (1949). The mathematical theory of communication. Philos. Rev. 93 (3), 31–32. doi: 10.1063/1.3067010

[B43] Shannon C. E. Weaver W. Wiener N. (1950). The mathematical theory of Communication. Physis Today. 9 (3), 31–32. doi: 10.1063/1.3067010.

[B44] Shi M. Wang Y. Duan T. Qian X. Zeng T. Zhang D. (2020). *In situ* glacial survival maintains high genetic diversity of *Mussaenda kwangtungensis* on continental islands in subtropical China. Ecol. Evol. 10, 11304–11321. doi: 10.1002/ece3.6768 33144966 PMC7593160

[B45] Slatkin M. Barton N. H. (1989). A comparison of three indirect methods for estimating average levels of gene flow. Evolution 43, 1349–1368. doi: 10.2307/2409452 28564250

[B46] Wang M. Zhang X. Zhang Y. Xiao M. (2022). Prevalence and genetic analysis of thalassemia and hemoglobinopathy in different ethnic groups and regions in hainan island, Southeast China. Front. Genet. 13. doi: 10.3389/fgene.2022.874624 PMC9245582 35783269

[B47] Wei S. X. Liang R. L. Lin J. Y. He Y. H. Jiang Y. (2020). Geographical distribution and community characteristics of *Albizia odoratissima* in China. Guangxi For Sci. 49, 71–75. doi: 10.19692/j.cnki.gfs.2020.01.014

[B48] Wickham H. (2016). ggplot2: elegant graphics for data analysis (New York: Springer-Verlag New York). doi: 10.1007/978-3-319-24277-4

[B49] Wright S. (1951). The genetic structure of populations. Ann. Eugen 15, 323–354. doi: 10.1111/j.1469-1809.1949.tb02451.x 24540312

[B50] Wright S. (1978). Variability within and among natural populations (Chicago: The Univ. Of Chicago Press).

[B51] Xia M. (1999). Research progress of genetic diversity. Chin. J. Ecol. (3), 59–65. Available at: http://www.cje.net.cn/EN/Y1999/V/I3/59.

[B52] Yeh F. C. Yang R. C. Boyle T. (1999). POPGENE version1.32, microsoft window-bass software for population genetic analysis: a quick user’s guide university of alberta, center for international forestry research (Canada: Alberta).

[B53] Yuan T. X. Huang Y. Q. Liang R. L. (2011). Main native tree species in Guangxi (Nanning: Guangxi Science and Technology Press), 149.

[B54] Zhu H. (2016). Biogeographical evidences help revealing the origin of Hainan Island. PloS One 11, e0151941. doi: 10.1371/journal.pone.0151941 27055236 PMC4824349

